# Serotonin and the ventilatory effects of etonogestrel, a gonane progestin, in a murine model of congenital central hypoventilation syndrome

**DOI:** 10.3389/fendo.2023.1077798

**Published:** 2023-02-21

**Authors:** Alexis Casciato, Lola Bianchi, Manon Reverdy, Fanny Joubert, Roman Delucenay-Clarke, Sandrine Parrot, Nélina Ramanantsoa, Eléonore Sizun, Boris Matrot, Christian Straus, Thomas Similowski, Florence Cayetanot, Laurence Bodineau

**Affiliations:** ^1^ Sorbonne Université, Inserm, UMR_S1158 Neurophysiologie Respiratoire Expérimentale et Clinique, Paris, France; ^2^ Centre de Recherche en Neurosciences, NeuroDialyTics, Bron, France; ^3^ Université de Paris, NeuroDiderot, Inserm, Paris, France

**Keywords:** CO_2_/H^+^ chemosensitivity, central breathing disorder, progestin, mouse model, serotoninergic systems

## Abstract

**Introduction:**

Congenital Central Hypoventilation Syndrome, a rare disease caused by *PHOX2B* mutation, is associated with absent or blunted CO_2_/H^+^ chemosensitivity due to the dysfunction of PHOX2B neurons of the retrotrapezoid nucleus. No pharmacological treatment is available. Clinical observations have reported non-systematic CO_2_/H^+^ chemosensitivity recovery under desogestrel.

**Methods:**

Here, we used a preclinical model of Congenital Central Hypoventilation Syndrome, the retrotrapezoid nucleus conditional *Phox2b* mutant mouse, to investigate whether etonogestrel, the active metabolite of desogestrel, led to a restoration of chemosensitivity by acting on serotonin neurons known to be sensitive to etonogestrel, or retrotrapezoid nucleus PHOX2B residual cells that persist despite the mutation. The influence of etonogestrel on respiratory variables under hypercapnia was investigated using whole-body plethysmographic recording. The effect of etonogestrel, alone or combined with serotonin drugs, on the respiratory rhythm of medullary-spinal cord preparations from *Phox2b* mutants and wildtype mice was analyzed under metabolic acidosis. c-FOS, serotonin and PHOX2B were immunodetected. Serotonin metabolic pathways were characterized in the *medulla oblongata* by ultra-high-performance liquid chromatography.

**Results:**

We observed etonogestrel restored chemosensitivity in *Phox2b* mutants in a non-systematic way. Histological differences between *Phox2b* mutants with restored chemosensitivity and *Phox2b* mutant without restored chemosensitivity indicated greater activation of serotonin neurons of the *raphe obscurus* nucleus but no effect on retrotrapezoid nucleus PHOX2B residual cells. Finally, the increase in serotonergic signaling by the fluoxetine application modulated the respiratory effect of etonogestrel differently between *Phox2b* mutant mice and their WT littermates or WT OF1 mice, a result which parallels with differences in the functional state of serotonergic metabolic pathways between these different mice.

**Discussion:**

Our work thus highlights that serotonin systems were critically important for the occurrence of an etonogestrel-restoration, an element to consider in potential therapeutic intervention in Congenital Central Hypoventilation Syndrome patients.

## Introduction

Congenital central hypoventilation syndrome (CCHS; also named Ondine’s curse) is a rare life-threatening sleep-related central hypoventilation (incidence 1/148,000–1/200,000 live births) associated with an absent or blunted respiratory response to hypercapnia (1). CCHS is associated with an autosomal dominant mutation in the paired-like homeobox 2B gene (*PHOX2B*) which encodes a highly conserved homeodomain transcription factor (2, 3). *PHOX2B* is considered as the disease-defining gene in CCHS (4). The most common mutations are the addition of trinucleotides encoding alanine in exon 3, *i.e.* polyalanine mutations, and 7-alanine expansion is the most frequent *(PHOX2B^27Ala/+^*) (2, 3). Other rare mutations, grouped as non-polyalanine, correspond to missense, frameshift, nonsense and stop codon mutations (3). Specific loss of PHOX2B cells in the retrotrapezoid nucleus (RTN) is the primary neuroanatomical defect observed in *Phox2b^27Ala/+^* mice and respiratory defects in CCHS are at least partly attributed to loss or dysfunction of CO_2_/H^+^ chemosensory PHOX2B neurons of the RTN (4–7). In the absence of effective curative treatment, patients are placed on assisted ventilation for life, at least when sleeping (8), and insufficient ventilatory support can expose patients to neural damage and impair their quality of life (4, 9).

We previously reported that two adult women with CCHS recovered CO_2_/H^+^ chemosensitivity concomitantly with the administration of desogestrel for contraception (10). This suggests that desogestrel or its biologically active metabolite, the 3-ketodesogestrel (etonogestrel), could activate or over-activate the residual CO_2_/H^+^ chemosensitivity present in certain CCHS patients (11), an effect which was not provoked by the upsurge of progesterone during pregnancy (12). This observation undermined the prevailing view that respiratory symptoms in CCHS are irreversible, and paved the way for treatment research. Unfortunately, the recovery of chemosensitivity under desogestrel was not confirmed in a third patient (13), suggesting that effect in CCHS patients was contingent on the status of yet unknown respiratory pathways. Such a target could be medullary serotonin (5-HT) neurons, which we have recently shown to express the activation marker *c-Fos* under etonogestrel in rodent models free from CCHS (14, 15). Another possible explanation could be that etonogestrel acts on PHOX2B residual cells by decreasing the level of toxic cellular mutant PHOX2B protein, as recently reported in neuroblastoma cell lines (16, 17).

Bearing in mind that desogestrel can induce recovery of CO_2_/H^+^ chemosensitivity in CCHS patients (10) and assuming that respiratory defects in CCHS patients are at least partly attributed to alteration in RTN PHOX2B cells (5, 6), we postulated first that etonogestrel would induce a restoration of CO_2_/H^+^ chemosensitivity in RTN conditional *Phox2b* mutant mice, and second that it would act either on 5-HT medullary neurons, on residual RTN PHOX2B cells (6), or both. To validate our hypotheses, we combined pharmacological applications, whole-body plethysmographic recordings on adult mice, electrophysiological recordings on *ex vivo* preparations, functional and phenotypic histology, and ultra-high-performance liquid chromatography. We chose to work under acute exposure conditions as our previous data showed that etonogestrel stimulated the baseline respiratory drive under these conditions in the same proportions as observed in CCHS patients (14, 15).

## Methods

### Ethical approval

Experiments were carried out in accordance with Directive 2010/63/EU of the European Parliament and of the Council of 22 September 2010 French law (2013/118). Protocols were approved by Charles Darwin Ethics Committee for Animal Experimentation (Ce5/2011/05; APAFIS#14259-2018032518034654v3 and #2210-2015100812195835v2) and all efforts were made to minimize the number of animals used and their suffering.

### Animals

Experiments were performed on adult (5-7 weeks old) and newborn (0-4 days old) mice: *Egr2^cre/+^; Phox2b^27Ala/+^* mutant mice (hereafter termed *Phox2b* mutants; adult 19.3 ± 0.7 g, n=14 and newborn 1.2 ± 0.2 g, n=106), wildtype littermates (adult 21.2 ± 0.8 g, n=7 and newborn 1.2 ± 0.2 g, n=123) and wildtype Swiss OF1 strain (newborn 2.2 ± 0.1 g, n=137; Charles River laboratories, L’Arbresle, France).

Mutant mice expressing a Congenital Central Hypoventilation Syndrome-causing expansion of the 20-residue poly-alanine stretch in *Phox2b* were generated by crossing *Egr2^cre/+^* males with *Phox2b^20-27Ala/20-27Ala^* females (6, 18). Genotyping was done on tail DNA to identify mutants and wildtype littermates. To detect *cre* for mutant mice identification, the primers AAATTTGCCTGCATTACCG and ATGTTTAGCTGG CCCAAATG were used, yielding a band of 250 pb.

### Drugs

Etonogestrel (3-ketodesogestrel and fluoxetine were purchased from Sigma-Aldrich (Saint-Quentin Fallavier, France). For *in vivo* experiments, etonogestrel was dissolved in oil. For *ex vivo* experiments, fluoxetine was prepared in saline and for etonogestrel was dissolved in dimethylsulfoxide with a final concentration dimethylsulfoxide at 0.01%.

#### Whole body plethysmography on adult mice

Male mice were acclimatized to plethysmograph chambers (DSI Buxco^®^ Multi-function Bias Flow, Harvard Bioscience, Holliston, USA) for at least one hour during 4 consecutive days. On the day of the experiment, respiratory variables were recorded while mice were quiet (after 20 min in the chamber). Normocapnic (21%O_2_ and 79%N_2_) and hypercapnic (8%CO_2_, 21%O_2_, 71%N_2_) gas mixtures were produced by a gas mixing device (ALICAT, RS-232 Multi Drop model BB9, Tucson, Arizona, USA; software Flowvision 1.0.16.0) and delivered continuously at 0.5 l.min^-1^. The recording session was as follows: 10 min in normocapnia followed by 10 min in hypercapnia. The plots were analyzed in order to evaluate the respiratory frequency (fR, respiratory cycles. min^−1^), tidal volume (VT) normalized as the ratio of VT divided by body weight (VT, μl.g^−1^) and minute ventilation (VE, ml.g^−1^. min^−1^).

*Phox2b* mutants received either 10^-4^ mg.kg^-1^ of etonogestrel *per os* (n=7) or solvent (n=7) 2 hours before measurements of respiratory variables. This protocol for administration was chosen on the basis of our previous work on mice free from CCHS (14). Wildtype littermate (n=8) received the solvent 2 hours before the measurements.

#### *Pharmacology on ex vivo* medullary-spinal cord preparation

##### Medullary-spinal cord preparations

Newborn mice were placed under deep cold anesthesia (19) and medullary-spinal cord preparations were dissected out, as previously described (14, 20, 21). The rostral section was made at the level of the anterior inferior cerebellar arteries just caudal to the eighth cranial nerve exit points. The caudal section was made between the seventh and the eighth cervical spinal roots. *Ex vivo* preparations were placed in a recording chamber with the ventral surface facing upward. They were superfused with artificial cerebrospinal fluid (in mM: 129 mM NaCl, 3.35 mM KCl, 1.26 CaCl_2_, 1.15 mM MgCl_2_, 0.58 mM NaH_2_PO_4_, 30 mM Glucose and NaHCO_3_ at various concentrations depending on the experimental condition (22) maintained at 27 ± 1°C, saturated with O_2_, and adjusted to the appropriate pH by bubbling with 95% O_2_ and 5% CO_2_. As molecular sensors detecting H^+^ and CO_2_ changes are described as sensitive to an increased concentration of H^+^, we performed pH variation of artificial cerebrospinal fluid to mimic physiological consequences of an increase in CO_2_ (7, 23, 24) (25). Normal-pH artificial cerebrospinal fluid (pH 7.4) and metabolic acidosis artificial cerebrospinal fluid (pH 7.23) differed in terms of NaHCO_3_ concentration (21 mM and 15 mM, respectively) (15, 20, 22, 23). Electrical activity of the fourth cervical ventral nerve root (C4) was recorded using a suction electrode, filtered (300-1000 Hz), amplified (x10000 Grass P511 AC Amplifier), integrated (Dual channel integrator, University of Chicago), and digitized through a Spike 2 data analysis system (CED micro 1401; Cambridge Electronic Design) with a sampling frequency of 2500 Hz. Respiratory-like rhythm (respiratory frequency, fR) was defined as the burst frequency recorded on the fourth cervical ventral nerve root for 1 minute (burst.min^-1^) (15, 20, 22, 23).

**Figure 1:**
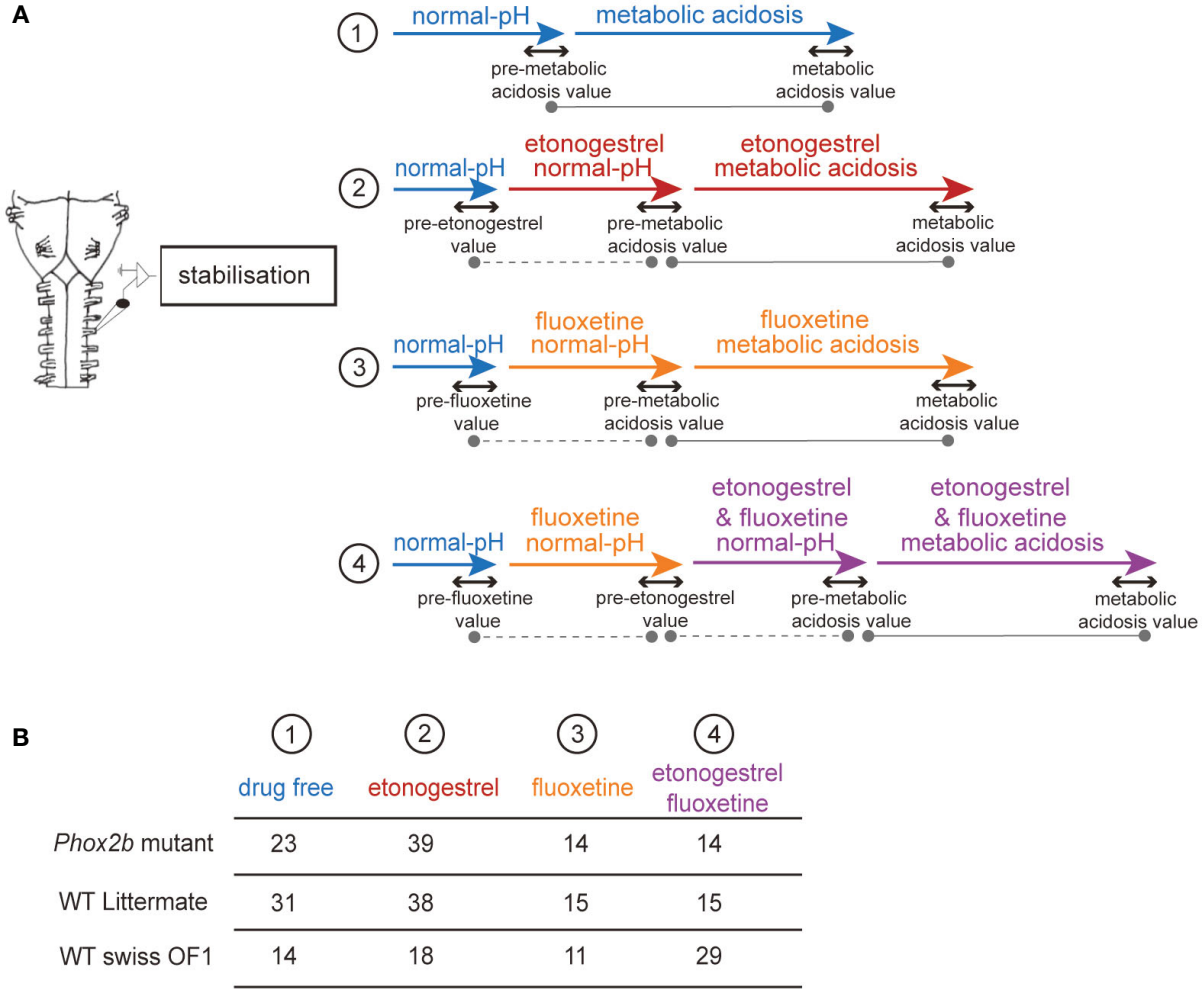
Schematic representation of the pharmacological protocols used. Flowchart of the protocols used in *ex vivo* medulla-spinal cord preparations from *Phox2b* mutants, wildtype littermates and wildtype Swiss OF1 mice **(A)**. Briefly, after a period allowing fR to stabilize, preparations were subjected to one of four protocols: 1) preparations were exposed to drug-free normal-pH and metabolic acidosis conditions, 2) preparations were exposed to drug-free normal-pH followed by exposure to etonogestrel under normal-pH and then metabolic acidosis conditions, 3) preparations were exposed to drug-free normal-pH followed by exposure to fluoxetine under normal-pH and then metabolic acidosis conditions, 4) preparations were exposed to drug-free normal-pH, then to fluoxetine under normal-pH followed by etonogestrel associated with fluoxetine under normal-pH and then metabolic acidosis conditions. Table indicating the number of *ex vivo* preparations used for each protocol in *Phox2b* mutants, wildtype littermates and wildtype Swiss OF1 mice **(B)**. The dotted grey lines represent the comparison made to determine the influence of a drug in normal-pH. The grey lines represent the comparison made to determine the response to metabolic acidosis under drug-free or drug exposure conditions.

After surgery, preparations were routinely allowed to stabilize for 30 minutes in normal-pH free of drug (**Figure 1**). Consistent with previous reports about the respiratory effect of etonogestrel or other steroids and 5-HT drugs on newborn rodents less than 4 days old, we pooled data obtained from males and females (14, 15, 26–28). Baseline values were defined as the mean value during the last 5 minutes of this stabilization period. Then, a given preparation was exposed to a given pharmacological protocol consisting of 20 minutes of drug under normal-pH conditions, followed by exposure to metabolic acidosis under the same drug for 30 minutes, followed by a 30-minute washout period. To evaluate the influence of a drug under normal-pH conditions, the last five minutes of drug exposure was compared to the five minutes preceding the drug exposure (**Figure 1**). The effect of a drug on the respiratory metabolic acidosis response was assessed by comparing the last five minutes before metabolic acidosis (pre-metabolic value) to the last five minutes of the metabolic acidosis (**Figure 1**). To compare between experimental conditions (animals and drugs), we normalized fR values during metabolic acidosis to the value of the last 5 minutes before metabolic acidosis. In set of mutant preparations, some showed an increase in their respiratory-like rhythm under metabolic acidosis and others not. As it has been done in similar conditions (29), we designated two populations defined by the ability (“acidosis-responder”) or failure (“acidosis-non-responder”) to increase their respiratory-like rhythm by at least 10%.

#### Pharmacological exposure to etonogestrel on Phox2b mutants and wildtype littermates

*Phox2b* mutant (n=23) and wildtype littermate (n=31) preparations were exposed only to the drug-free metabolic acidosis protocol to assess whether they showed a respiratory response to metabolic acidosis.

Based on previous work (14, 15), to determine the respiratory influence of etonogestrel in normal-pH conditions and the respiratory response to metabolic acidosis, 39 preparations of *Phox2b* mutants and 38 preparations of wildtype littermates were exposed to 5.10^-2^ µM of etonogestrel.

#### Pharmacological exposure to etonogestrel co-applied with fluoxetine in OF1 mice, Phox2b mutants and wildtype littermates

We investigated whether the combination of etonogestrel with a selective serotonin reuptake inhibitor, fluoxetine, induced a respiratory benefit. As the respiratory influence of fluoxetine has been shown to be variable according to its concentration in rodent newborns (28, 30, 31), we tested different concentrations to determine a dose that did not cause respiratory depression in OF1 mice. Preparations were superfused for 20 minutes at normal-pH with fluoxetine at 6.25 µM (n=10), 12,5 µM (n=11), 25 µM (n=10), 50 µM (n=4) and 100 µM (n=4); 12.5 µM was retained (see results). Second, preparations were exposed to either 5.10^-2^ µM etonogestrel alone (n=18), 12.5 µM fluoxetine alone (n=11) or both 5.10^-2^ µM etonogestrel and 12.5 µM fluoxetine (n=29) for 20 minutes at normal-pH, followed by 30 minutes under metabolic acidosis with the considered drug and finally 30 minutes under normal-pH free of drug (**Figure 1**).

Given the results obtained in OF1 mice preparations, we investigated a respiratory benefit to the combination of etonogestrel with fluoxetine in *Phox2b* mutants. First, preparations from 10 *Phox2b* mutants and 15 wildtype littermates were exposed to 12.5 µM of fluoxetine at normal-pH. At this concentration, fluoxetine decreased the fR of *Phox2b* mutant mice. We therefore decided to test 3.125 µM (n=14 *Phox2b* and n=15 wildtype littermates) and 6.25 µM (n=12 *Phox2b* and n=16 wildtype littermates) of fluoxetine. 3.125 µM fluoxetine was retained (see results). Second, *Phox2b* mutant (n=14) and wildtype littermates (n=15) preparations were co-exposed to 5.10^-2^ µM etonogestrel and 3.125 µM fluoxetine under normal-pH and metabolic acidosis (**Figure 1**), followed by a washout with normal-pH free of drug.

### Histology

Immunohistological investigations were performed on acidosis-responder and acidosis-non-responder *Phox2b* mutants exposed to 5.10^-2^ µM etonogestrel as described above with the aim of revealing the cell populations responsible for the different respiratory behavior between the two types of mutants. After the 30-minute period of metabolic acidosis exposure under etonogestrel, preparations were fixed by immersion in 4% paraformaldehyde in 0.1 M phosphate buffer (pH 7.4) for 72 h at 4°C. Then, they were cryoprotected for 48 hours in 0.1 M phosphate buffer containing 30% sucrose and stored at -20°C until use. Free-floating coronal sections (30 µm) obtained using a cryostat (Leica CM 1510S) were used for immunohistological investigations.

Dual immunohistochemical detections of c-FOS and 5-HT were processed. First, sections were incubated with a rabbit polyclonal antibody against c-FOS (sc-253; Santa Cruz Biotechnology Inc., CA, USA, 1:8000, in 1% bovine serum albumin, BSA; 48 h, 4°C), then with a biotinylated goat anti-rabbit IgG antibody (BA-1000, Vector Laboratories, Burlington, Canada; 1:500; in 1% BSA; 2 h, room temperature), and finally an avidin-biotin-peroxidase complex (ABC kit standard, PK-6100, Vectastain, Vector Laboratories, Burlingame, CA, USA; 1:250; 1 h). Peroxidase activity was detected using a solution containing 0.02% 3,3’-diaminobenzidine tetrahydrochloride, 0.04% nickel ammonium sulfate and 0.01% H_2_O_2_ in 0.05 m Tris-HCl buffer (pH 7.6), which results in a blue/grey chromogen in the nucleus. Second, sections were incubated with a rabbit polyclonal antibody against 5-HT (S8305, Sigma-Aldrich, Saint-Quentin Fallavier, France; 1:20000, in 1% BSA; 48 h, 4°C). Sections were then incubated with biotinylated goat anti-rabbit (BA-1000, Vector Laboratories, Burlington, Canada; 1:500 in 1% BSA; 2 h, room temperature), and then ABC (1:250; 1 h). Peroxidase activity was detected using a solution containing 0.02% 3,3’-diaminobenzidine tetrahydrochloride and 0.01% H_2_O_2_ in 0.05 m Tris-HCl buffer (pH 7.6), which results in a brown chromogen in the cytoplasm very clearly distinguishable from blue/grey staining in the nucleus for c-FOS. Some sections were processed in parallel, but with the omission of primary or secondary antibodies. No labelling was observed in these conditions. All sections were mounted in sequential caudo-rostral order on silanized slides, air-dried, and coverslipped with EUKITT (Bio Optica, Milan, Italy). Sections were photographed with a digital camera (Leica DFC450C, Leica Microsystems, Heidelberg, Germany) coupled to a microscope (Leica DM2000; Leica Microsystems, Heidelberg, Germany), using the software Leica Application Suite (L.A.S V4.5). c-FOS, 5-HT, and c-FOS/5-HT immunolabeled cells were counted by an investigator blinded to samples. Analysed medullary structures involved in elaboration or adaptation of the respiratory drive were localized using standard landmarks (32, 33). c-FOS positive cells were counted in commissural and medial parts of the nucleus of the solitary tract (c/mNTS), in the ventrolateral medullary reticular nucleus (VLM), a neuronal column ventral to nucleus ambiguus, extending from pyramidal decussation to caudal edge of facial nucleus, that contains the ventral respiratory column in medullary raphe nuclei *i.e.* the raphe *obscurus* (ROb), the raphe *pallidus* (RPa) and in the ventral medullary surface. A distinction was made between the caudal part of the raphe *pallidus* and raphe *obscurus* (from the pyramidal decussation to the rostral edge of the inferior olive, cRPa and cROb) and their rostral part (from the rostral edge of the inferior olives to the rostral edge of the facial nucleus, rRPa and rROb) (14). In the ventral medullary surface, the c-FOS cell count was made in ventral position under the facial nucleus and also immediately caudal to it to cover the region containing the retrotrapezoid nucleus and the parafacial respiratory group (5, 34, 35).

Dual detections of c-FOS and PHOX2B by immunohistofluorescence were performed. Sections were co-incubated with a rabbit polyclonal antibody against c-FOS (sc-253; Santa Cruz Biotechnology Inc., CA, USA, 1:8000, in 1%, BSA; 48 h, 4°C) and a mouse monoclonal antibody against PHOX2B (sc-376993; Santa Cruz Biotechnology Inc., CA, USA, 1:1000, in 1%, BSA; 48 h, 4°C). Sections were then incubated with an Alexa Fluor 488-labeled donkey anti-rabbit antibody (Molecular Probes, Eugene, OR) and an Alexa Fluor 555-labeled goat anti-mouse antibody (Invitrogen, Thermo Fisher Scientific) concomitantly with DAPI at 1:1000 (Immunochemistry technologies), 2 hours at room temperature. They were then washed, mounted in sequential caudo-rostral order on silanized slides, air-dried, and coverslipped using Fluoromount G (Fluoromount Aqueous Mounting Medium, Sigma-Aldrich, Saint-Quentin Fallavier, France). Sections were photographed with a digital camera (Leica DFC450C, Leica Microsystems, Heidelberg, Germany) coupled to a microscope (Leica DM2000; Leica Microsystems, Heidelberg, Germany), using the software Leica Application Suite (L.A.S V4.5). Microphotographies were then processed with the Image J platform and the c-FOS, PHOX2B and c-FOS/PHOX2B cells were counted by an investigator blinded to samples in the caudal retrotrapezoid nucleus, parafacial retrotrapezoid nucleus, and parafacial respiratory group.

### Ultra high-performance liquid chromatography neurotransmitter analyses

The levels of 5-HT, its precursor 5-hydroxytryptophan (5-HTP) and its metabolite 5-hydroxyindole acetic acid (5-HIAA) were measured by ultra high-performance liquid chromatography in the medulla oblongata of *Phox2b* mutants (n=8), wildtype littermates (n=8) and OF1 mice (n=10). After rapid cold surgery, the medulla oblongata was taken and kept at -80°C until analyses (36). Before ultra high-performance liquid chromatography, tissues were ground by sonication and centrifuged, the supernatant was used to quantify 5-HT and its related compounds, 5-HTP and 5-HIAA. Tissue analyte levels were quantified by ultra high-performance liquid chromatography coupled with electrochemical detection. The ultra high-performance liquid chromatography system consisted of a degasser (Prominence), a high-pressure (LC-30 AD pump) and an autosampler (SIL-30AC Shimadzu). Separations were performed using a 100 x 2.1 mm Kinetex C18 core-shell 2.6 µm column (00D-4462-AN, Phenomenex) equipped with an Ultra Security Guard (AJ0-8782, Phenomenex) as a precolumn. The mobile phase (70 mmol/L potassium phosphate containing 0.1 mmol/L EDTA, 6.6 mmol/L octane sulfonate, 3.1 mmol/L triethylamine, 10% methanol, pH adjusted to 3.12 with 1 mmol/L citric acid, filtered), was pumped at a flow rate of 0.4 mL/min. Analytes were detected at an oxidation potential of 800 mV versus the reference electrode. Chromatograms were acquired at a rate of 10 Hz using Lab Solutions software for 17 minutes per sample. All the samples had to be diluted 3-fold in the extraction solution to properly quantify 5-HIAA. Concentrations of analytes were determined by comparison of chromatographic peak areas with calibration curves derived from a mixture of synthetic standards. Final concentrations were expressed as g of analytes per medulla oblongata and 5-HIAA/5-HT and 5-HT/5-HTP ratios were calculated.

### Statistics

Data were analyzed with GraphPad (GraphPad Prism9, San Diego, California, United-States). Normality of data distribution for fR, VT and VE, for cells immunolabelled c-FOS, 5-HT, c-FOS/5-HT, PHOX2B, c-FOS/PHOX2B, for concentrations of 5-HT, 5-HTP and 5-HIAA, and for 5-HT/5-HTP and 5-HIAA/5-HT ratios were assessed using the d’Agostino and Pearson omnibus normality test. Depending on whether the distribution was normal or not, data were expressed as mean ± standard deviation or median and interquartile range [Q1; Q3] and parametric or non-parametric tests were performed: within a group of preparation, in paired conditions, paired t-test, Wilcoxon signed rank test, one-way analysis of variance, or Friedman test followed by Dunn’s or Benjamini, Krieger and Yekutieli’s multiple comparison test; between two groups, in unpaired conditions, unpaired t test, Mann & Whitney test and two-way analysis of variance followed by Benjamini, Krieger and Yekutieli’s multiple comparison test in unpaired conditions; between more than two groups, one way analysis of variance or Friedman test followed by Benjamini, Krieger and Yekutieli’s multiple comparison test. Percentages of *Phox2b* mutant mice acidosis-responder or acidosis-non-responder with or without etonogestrel were compared by Fisher’s exact test. Differences were considered to significant if p < 0.05.

## Results

### Recovery of the CO_2_/H^+^ chemosensitivity by etonogestrel in *Phox2b* mutants

#### Etonogestrel leads to the restoration in adult Phox2b mutant mice of a response to hypercapnia similar to that of WT littermate

**Figure 2:**
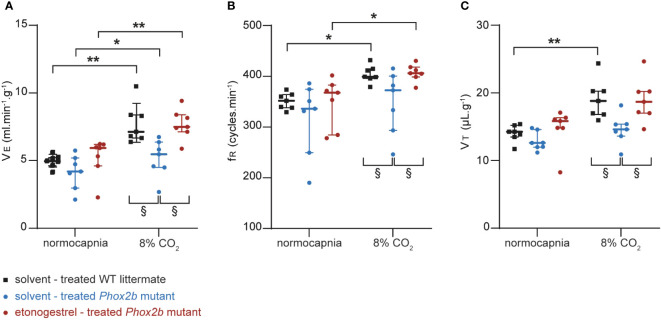
Effect of etonogestrel on the ventilation in adult male *Phox2b* mutant mice. Scatter plots with median and interquartile [Q1; Q3] superimposed of the minute ventilation (VE; **A**), respiratory frequency (fR; **B**) and tidal volume (VT; **C**). *Indicates a significant difference in VE, fR or VT relative to normocapnic values (*p < 0.05, **p < 0.01; one-way analysis of variance or Friedman test followed by Benjamini, Krieger and Yekutieli’s multiple comparison test). ^§^ Indicates a significant difference between mice groups ^§^ p < 0.05; two-way analysis of variance test followed by Benjamini, Krieger and Yekutieli’s multiple comparison test).

Adult male *Phox2b* mutant mice had a normocapnic baseline VE comparable to that of WT littermates (p=0.42; **Figure 2A**). As already reported (6), we observed that adult *Phox2b* mutants increased their VE under hypercapnia (p<0.03) but significantly less than what is observed in WT littermates (p<0.03; **Figure 2A**). Note that both fR and VT of *Phox2b* mutant mice tended to increase without this being significant (p=0.17 and p=0.32 respectively; **Figures 2B, C**).

Etonogestrel-treated *Phox2b* mutants showed an increase in VE induced by hypercapnia (p<0.003; **Figure 2A**). This increase was enhanced compared to solvent-treated *Phox2b* mutants (p<0.03; **Figure 2A**), but similar to that observed in WT littermates (p=0.96; **Figure 2A**). The increase in VE in etonogestrel-treated *Phox2b* mutants was supported by a significant increase in fR (p<0.015; **Figure 2B**). Even if hypercapnia did not induce a significant increase in VT under progestin, the VT is significantly greater under etonogestrel than under solvent (p<0.015; **Figure 2C**).

#### Etonogestrel restores a response to metabolic acidosis in Phox2b mutant ex vivo preparations

**Figure 3:**
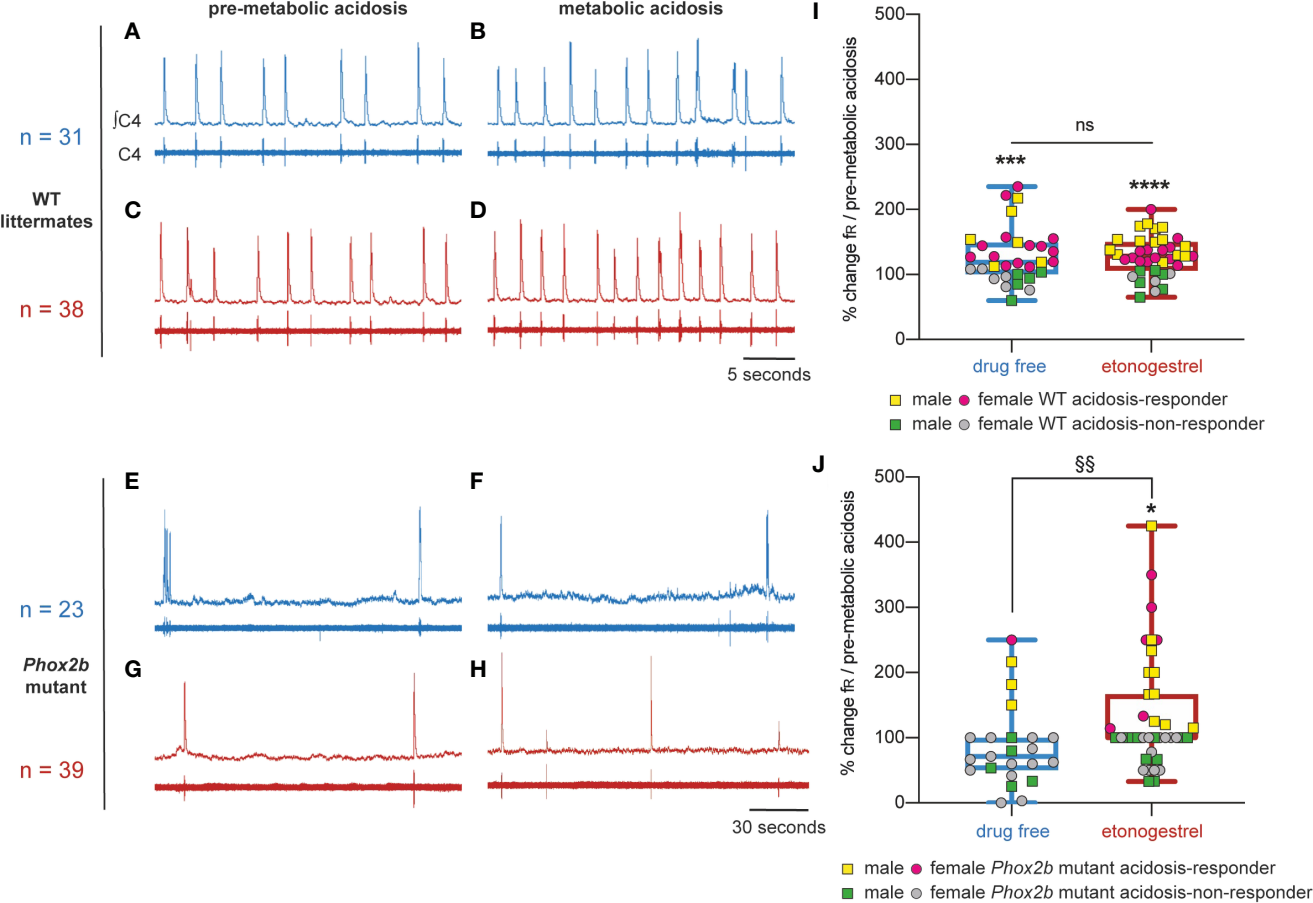
Effects of etonogestrel on central H^+^ chemosensitivity in *Phox2b* mutant mice. **(A–H)**, Individual phrenic activity traces (fourth cervical ventral nerve root, C4) and integrated C4 activity from medullary-spinal cord from wildtype littermates **(A–D)** and *Phox2b* mutant mice **(E–H)** preparations, under drug-free **(A, B, E, F)** or etonogestrel (**C, D, G, H;** 5.10^-2^ µM) exposure, in normal-pH (**A, C, E, G**; pre-metabolic acidosis) or metabolic acidosis conditions **(B, D, F, H)**. **(I, J),** Scatter plots with a superimposed box and whisker (median [Q1; Q3] and minimum and maximum values) showing the respiratory-like rhythm (respiratory frequency, fR) during the last five minutes of metabolic acidosis in percentage of pre-metabolic acidosis values under drug-free and etonogestrel exposure in wildtype littermates (**I**; n=31 and n=38 respectively) and *Phox2b* mutant mice (**J**; n=23 and n=39). Etonogestrel induced not only a restoration of the response to metabolic acidosis considering all preparations, but also an increase in the proportion of acidosis-responder (preparations displaying an increase in fR under metabolic acidosis compared to pre-metabolic acidosis values by at least 10%, p<0.05; Fisher’s exact test; **J**, yellow squares for males and pink circles for females). *Indicates a significant difference in fR relative to pre-metabolic acidosis values (*p < 0.05, ***p < 0.001, ****p < 0.0001; paired t-test or Wilcoxon test). ^§^Indicates a significant difference in *Phox2b* mutant mice between preparation exposed to metabolic acidosis with and without etonogestrel (^§§^p < 0.01; Mann-Whitney test). ∫C4, integrated activity of C4 ventral nerve root; C4, electrical activity of C4 ventral nerve root, WT, wildtype.

Preparations from *Phox2b* mutants displayed a much slowed respiratory-like rhythm in normal-pH, significantly lower than that of wildtype littermates (0.8 ± 0.1 cycles.min^-1^, n=98 *vs* 13.9 ± 0.8 cycles.min^-1^, n=115; p<0.0001). Under metabolic acidosis, wildtype littermates showed increased respiratory frequency (fR, +29%, p<0.001; **Figures 1**, **3A, B, I**). For all mutant preparations collected, there was no response to metabolic acidosis as previously described (6). Note that we pooled males and females because we did not observe significant gender differences (p=0.58) (**Figures 3E, F, J**). However, some preparations increased their respiratory-like rhythm and others not. As in similar conditions (29), we designated two populations defined by the ability (“acidosis-responder”) or failure (“acidosis-non-responder”) to increase their respiratory-like rhythm by at least 10%: 4 of the 23 preparations (17%) were thus considered as acidosis-responders (**Figures 3F, J**).

Under etonogestrel, while the respiratory-like rhythm of *Phox2b* mutant preparations was not modified in normal-pH (p=0.33) (**Figure 3E**), it significantly increased (+35%; p<0.001) under metabolic acidosis conditions (**Figures 1**, **3H, J**). As in the absence of progestin, there was no difference between males and females (p=0.58), so we pooled them. The proportion of acidosis-responder preparations was greater in presence of etonogestrel (16 of 39 preparations, 41% vs 17% without etonogestrel; p<0.05) than without the progestin (**Figure 3J**). In wildtype littermates preparations, etonogestrel led to a significant increase in fR in normal-pH compared to pre-etonogestrel values (16.4 ± 1.6 vs 13.0 ± 1.3 cycles.min^-1^, +29%, p=0.0003; **Figures 1**, **3C**), and did not modify the increase in respiratory-like rhythm induced by metabolic acidosis (+28%, 19.3 ± 1.4 cycles.min^-1^ vs 29% without etonogestrel; p<0.0001; **Figures 3D, I**).

### Difference in *c-Fos* expression under metabolic acidosis/etonogestrel between acidosis-responder and acidosis-non-responder *Phox2b* mutants

**Figure 4:**
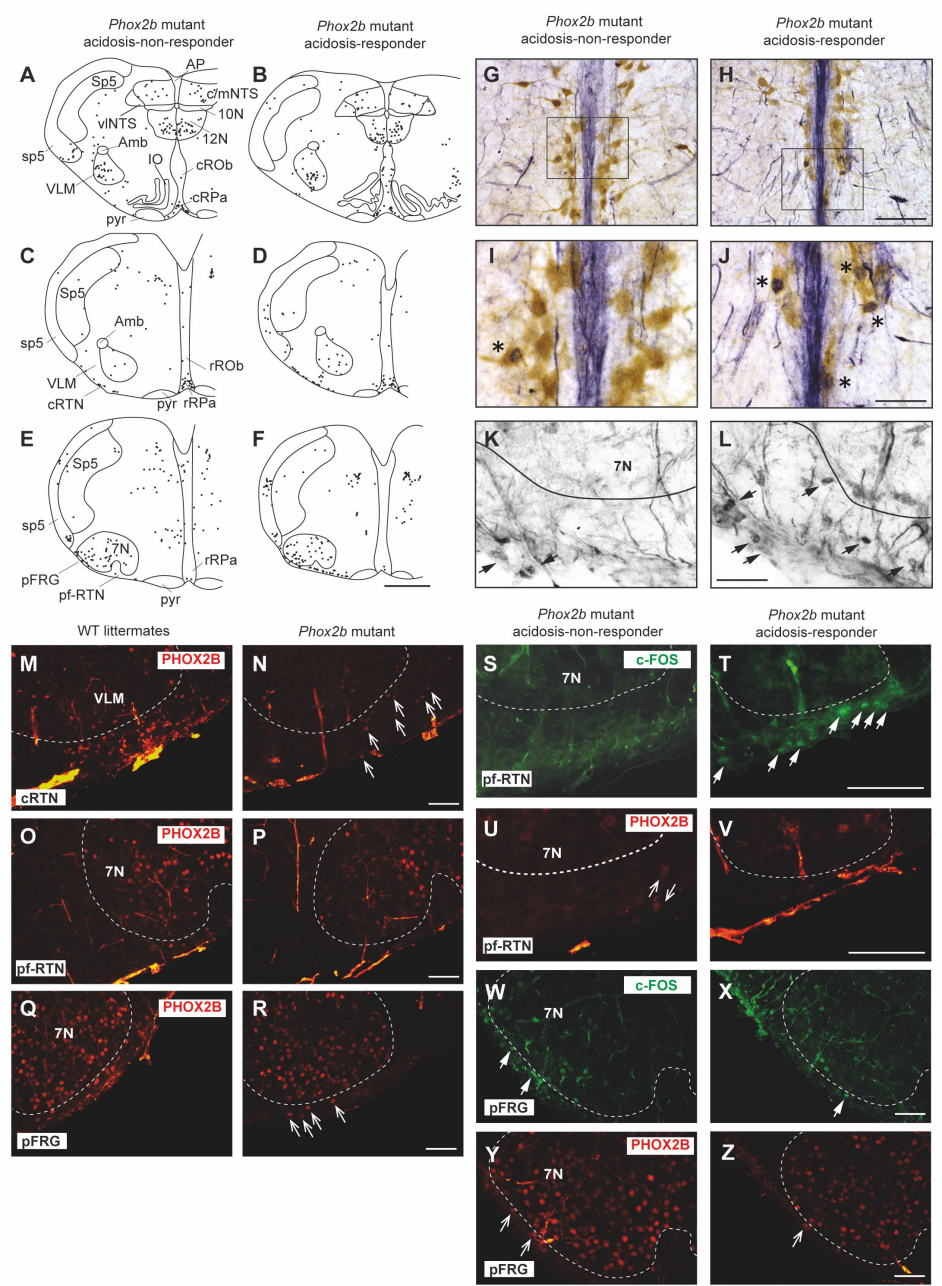
Comparison of the *c-fos* expression between acidosis-responder and acidosis-non-responder *Phox2b* mutant preparations under etonogestrel. Drawings of representative sections from the medulla oblongata at the caudal level (with inferior olives; **A**, **B**), intermediate level (between inferior olives and the facial nucleus; **C**, **D**) and rostral level (with the facial nucleus; **E**, **F**) illustrating the c-FOS distribution in non-acidosis responder **(A, C, E)** and acidosis-responder **(B, D, F)** *Phox2b* mutants under etonogestrel. Photomicrographs of c-FOS/5-HT immunoreactivities in the caudal ROb of acidosis-non responder **(G, I)** and acidosis-responder **(H**, **J)** *Phox2b* mutants. Photomicrographs in **(I**, **J)** correspond to enlargements of the outlined area in **(G, H)**; note the greater number of dual-labeled c-FOS/5-HT neurons (*) in acidosis-responder than in non-acidosis responder *Phox2b* mutants. Photomicrographs of c-FOS immunoreactivity in the ventral medullary surface, ventromedial to the facial nucleus in the pf-RTN **(K, L)** showing that the number of c-FOS positive neurons (solid black arrows) was greater in acidosis-responder than in non-acidosis-responder *Phox2b* mutants. Photomicrographs of immunofluorescence detection for PHOX2B in wildtype littermates **(M, O, Q)** and *Phox2b* mutants **(N, P, R)** in the ventral medullary surface, just below the caudal edge of the facial nucleus, in the cRTN **(M**, **N)**, ventromedial to the facial nucleus, in the pf-RTN **(O, P)** and ventrolateral below the facial nucleus, in the pFRG **(Q, R)**. In wildtype littermates, PHOX2B neurons are distributed in all three delimitations with the highest number of neurons in the cRTN as already described (5). In *Phox2b* mutants, the number of neurons was drastically reduced but some residual cells were still present as previously described (hollow white arrows) (6). Dual c-FOS **(S, T, W, X)** and PHOX2B **(U, V, Y, Z)** detections by immunofluorescence under the ventromedial part of the RTN (pf-RTN) in acidosis-non-responder **(S**, **U)** and acidosis-responder **(T, V)** *Phox2b* mutants and under the pFRG in acidosis-non-responder **(W**, **Y)** and acidosis-responder **(X, Z)** *Phox2b* mutants; note the presence of c-FOS positive (solid white arrows) but PHOX2B negative cells in acidosis-responder *Phox2b* mutants. Scale bar = 200 μm **(A–F)**, 100 µm **(M–R)**, 50 μm **(G, H)**, and 20 µm **(I, J, K, L, S, T, U, V)**. 7N, facial nucleus; 10N, dorsal motor nucleus of the vagus; 12N, hypoglossal nucleus; Amb, ambiguus nucleus; AP, area postrema; c/mNTS, commissural and median parts of the nucleus of the tractus solitarius; IO, inferior olives; VLM, ventrolateral medullary reticular nucleus; pFRG, parafacial respiratory group; pyr, pyramidal tract; ROb, raphe obscurus nucleus (caudal part—cROb, from pyramidal decussation to rostral edge of the inferior olive and rostral part—rROb, from rostral edge of the inferior olives to rostral edge of the facial nucleus); RPa, raphe pallidus nucleus (caudal part—cRPa, from pyramidal decussation to rostral edge of the inferior olive and rostral part—rRPa, from rostral edge of the inferior olives to rostral edge of the facial nucleus); RTN, retrotrapezoid nucleus (parafacial RTN—pf-RTN, in ventromedial position under the facial nucleus and caudal RTN—cRTN, immediately to the caudal edge of the facial nucleus); sp5, spinal trigeminal tract; Sp5, spinal trigeminal interpolaris nucleus; vlNTS, ventrolateral part of the nucleus of the tractus solitaries; VLM, ventrolateral medullary reticular nucleus; WT, wildtype. Note that the blue staining along the medial axis in **(G–J)** photomicrographs corresponds to background observed when c-FOS labeling was revealed with Ni-concentrated DAB solution so that the contrast between c-FOS-positive nuclei and 5-HT-positive soma could be clearly distinguished.

To identify the origin of the etonogestrel-associated recovery of H^+^ chemosensibility in *Phox2b* mutants, we compared the c-FOS number of cells in medullary respiratory areas between acidosis-responders (n=8) and acidosis-non-responders (n=10) (**Table 1**; **Figure 4**).

**Table 1:** c-FOS-positive number of cells in respiratory areas of the medulla oblongata in *Phox2b* mutant preparations under etonogestrel.

	acidosis-non-responder (n=8)	acidosis-responder (n=6)
c/mNTS	146.5 [121.1; 181.5]	106.5 [83.0; 178.0] ^ns^; p=0.242
cROb	2.0 [1.0; 7.5]	24.5 [9.5; 38] *; p=0.028
rROb	5.5 [1.3; 9.0]	10.5 [2.5; 50.8] ^ns^; p=0.342
cRPa	10.5 [7.0; 22.0]	25.5 [10.0; 35.0] ^ns^; p=0.157
rRPa	74.5 [40.0; 102.0]	57.0 [32.5; 152.0] ^ns^; p=0.457
VLM	263.5 [220.3; 318.5]	449.0 [275.3; 497.3] ^ns^; p=0.062
pf-RTN	16.0 [12.8; 29.5]	36.0 [29.8; 59.0] *; p=0.015
cRTN pFRG	75.0 [62.9; 95.4] 19.0 [15.8; 35.8]	49.8 [34.0; 95.8] ^ns^; p=0.141 29.0 [19.5; 45.5] ^ns^; p=0.289

Values as expressed as median number of total c-FOS-positive cells [Q1; Q3].

*Indicates a significant difference between acidosis-responder Phox2b mutant values compared with acidosis non-responder Phox2b mutant values; ns, non significant

c/mNTS, commissural and median part of the solitary tract nucleus; cROb, caudal part of the nucleus of the raphe obscurus (from the pyramidal decussation to the rostral edge of the inferior olive); rROb; rostral part of the nucleus of the raphe obscurus (from the rostral edge of the inferior olives to the rostral edge of the facial nucleus); cRPa, caudal part of nucleus of the raphe pallidus (from the pyramidal decussation to the rostral edge of the inferior olive); rRPa, rostral part of nucleus of the raphe pallidus (from the rostral edge of the inferior olives to the rostral edge of the facial nucleus); VLM, ventrolateral reticular nucleus of the medulla; pf-RTN, RTN in a ventromedial position under the facial; cRTN, RTN immediately caudal to the facial nucleus; pFRG, parafacial respiratory group nucleus.

**Figure 5:**
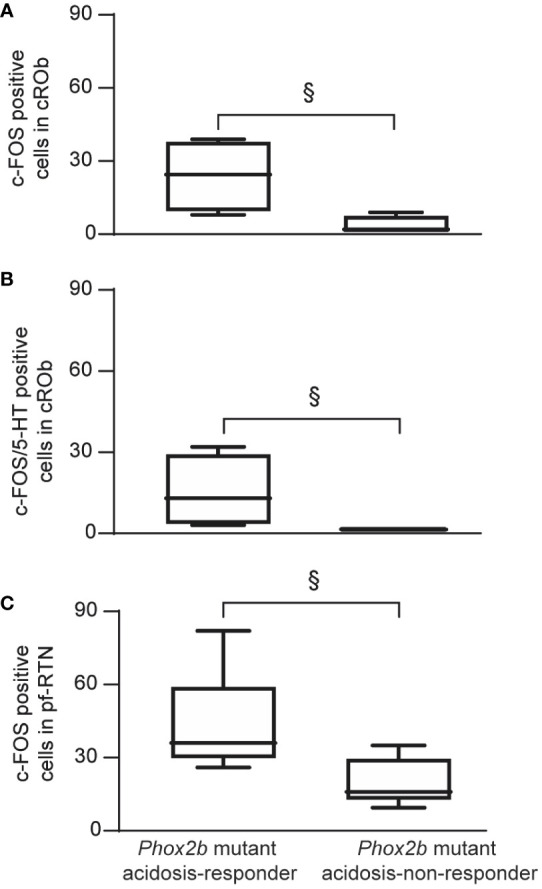
Graphical representation of the increase in number of c-FOS or c-FOS/5-HT positive cells in ROb and pf-RTN in *Phox2b* mutant acidosis-responder compared to acidosis non-responder under metabolic acidosis with etonogestrel. Box and whisker (median [Q1; Q3] and minimum and maximum values) showing the c-FOS **(A, C)** or c-FOS/5-HT **(B)** positive cells in the cROB **(A, B)** and pf-RTN **(C)**. (^§^p < 0.05; Mann & Whitney test). cROb (caudal part of the raphe obscurus nucleus; from pyramidal decussation to rostral edge of the inferior olive); pf-RTN (parafacial retrotrapezoid nucleus; in ventromedial position under the facial nucleus).

Under etonogestrel, acidosis-responder *Phox2b* mutants displayed a greater number of c-FOS cells in the *raphe obscurus* nucleus (ROb; **Table 1**; **Figures 4A, B**, **5A**). This increase concerned more particularly the part of the ROb at the rostro-caudal level delimited by the presence of inferior olives (**Table 1**; **Figures 4A, B**, **5A**). ROb contains different cell types, including 5-HT neurons (37, 38); we observed a significant increase in the number of doubly marked c-FOS/5-HT neurons of acidosis-responder *Phox2b* mutants compared to acidosis-non-responders (13.0 [3.5; 29.5] vs 1.0 [0.0; 2.0], p<0.05; **Figures 4G–J**, **5B**). Examination of the relationship between the number of c-FOS or c-FOS/5-HT neurons in the cROb and the level of metabolic acidosis-induced change in fR showed no correlation (Pearson correlation coefficient, r^2^= 0.25, p=0.21 and r^2^ = 0.14, p=0.37, respectively). In addition, it should be noted that the difference in c-FOS/5-HT cells in ROb was not due to a different number of 5-HT neurons (788.0 [523.0; 982.0] vs 977.0 [659.0; 1015.0], p>0.05 respectively), a finding that is aligned with the literature (5). In the adjacent *raphe pallidus* nucleus, we did not observe a significant difference either in the number of c-FOS (**Table 1**; **Figures 4A–F**) or c-FOS/5-HT neurons (32.5 [13.0; 44.5] vs 17.5 [11.3; 37.0], p>0.05 respectively) between acidosis-responder and acidosis-non-responder *Phox2b* mutants.

In line with published data (6), we found residual PHOX2B cells in the RTN of *Phox2b* mutants (**Figures 4M–R**). Their number was, expectedly, significantly lower (55.0 [39.0; 98.0]) than in wildtype littermates (291.0 [229.0; 326.0], n=3; p<0.0001). The RTN is described as a short column of relatively sparse neurons in the ventromedial position under the facial nucleus in its rostral part and also immediately to it in its caudal part (5, 35, 39), named respectively hereafter parafacial RTN (pf-RTN) and caudal RTN (cRTN). In the newborn, RTN is considered to be intertwined with the parafacial respiratory group (pFRG; expiratory generator) that is ventrolaterally below the facial nucleus and also contains PHOX2B cells (34, 40, 41). We thus examined pf-RTN and cRTN as defined above, and pFRG. We did not observe a significant difference in residual PHOX2B cells between acidosis-responder and acidosis-non-responder *Phox2b* mutants in pf-RTN (15 [7; 24] vs 13.5 [5.5; 21.5] respectively), cRTN (27 [17; 51] vs 16.5 [19.5; 33.5] respectively) (**Figures 4U, V, Y, Z**) and pFRG (15.5 [15; 23] vs 12 [5; 16.5] respectively). Yet, we found that acidosis-responder *Phox2b* mutants displayed an increase in the number of c-FOS cells in the pf-RTN (**Table 1**; **Figures 4E, F, K, L**, **5C**), but showed no difference at the level of cRTN and pFRG (**Table 1**; **Figures 4C–F, W, X**). Because there was virtually no c-FOS/PHOX2B cells in the pf-RTN, the increase in the number of c-FOS cells in pf-RTN was from PHOX2B-negative cells only (**Figures 4S–V**). Examination of the relationship between the number of c-FOS neurons in the pf-RTN and the level of metabolic acidosis-induced change in fR showed no correlation (Spearman correlation coefficient, r=0.58, p=0.08).

In other respiratory-related areas of the medulla oblongata, *i.e.* the ventrolateral reticular nucleus of the medulla (VLM), a part of the reticular formation that contains the ventral respiratory column and the commissural and median part of the nucleus of the solitary tract (c/mNTS), we did not observe any difference between acidosis-responder and acidosis-non-responder *Phox2b* mutants (**Table 1**; **Figures 4A–F**).

### Impact of 5-HT signaling on the respiratory response to metabolic acidosis under etonogestrel

Since 5-HT neurons of the ROb displayed an increase in *c-Fos* expression under metabolic acidosis/etonogestrel in acidosis-responder compare to acidosis-non-responder mutants, we sought to determine whether pharmacological modification of 5-HT signaling modulated the effect of etonogestrel.

#### An increase in 5-HT signaling associated with etonogestrel leads to a potentiation of the respiratory response to acidosis in wildtype Swiss OF1 mice

**Figure 6:**
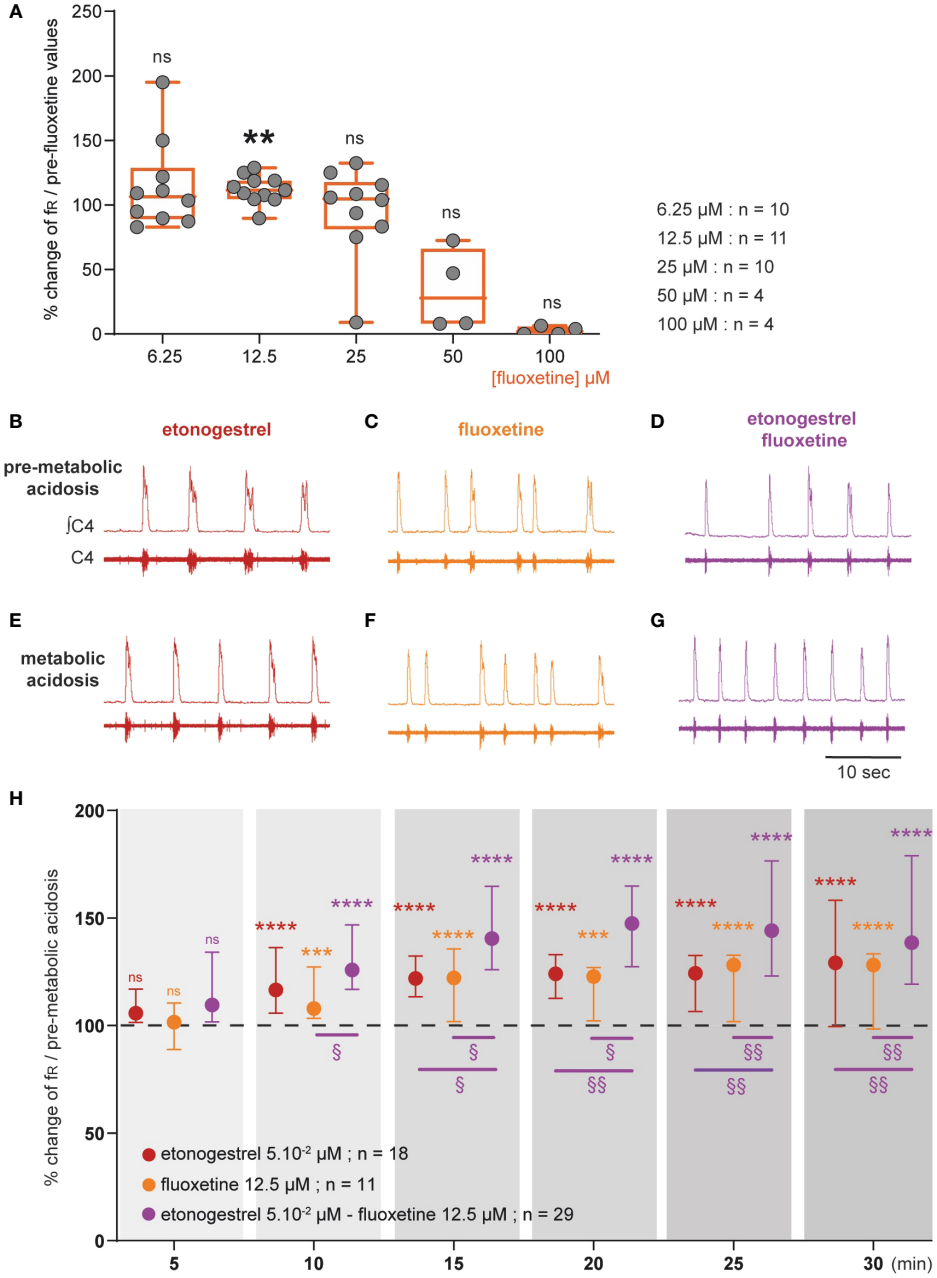
Effect of an increase in 5-HT signaling by fluoxetine on the respiratory response to metabolic acidosis under exposure to etonogestrel in OF1 mice. **(A)**, Scatter plots with a superimposed box and whisker plot (median [Q1; Q3]) showing percentage of change of fR under 6.25 (n=10), 12.5 (n=11), 25 (n=10), 50 (n=4) and 100 µM (n=4) of fluoxetine. **p < 0.01 indicates a significant change in fR relative to pre-fluoxetine values (paired t-test or Wilcoxon matched pairs signed rank test depending on the normality of data distribution). **(B–G)**, Individual phrenic activity traces (fourth cervical ventral nerve root, C4) and integrated C4 activity from medullary-spinal cord from OF1 mice in normal-pH (**B–D**; pre-metabolic acidosis) and metabolic acidosis **(E–G)** conditions, under etonogestrel **(B, E)**, fluoxetine **(C, F)**, or etonogestrel/fluoxetine **(D, G)** exposure. **(H)**, Median with interquartile range [Q1; Q3] illustrating the respiratory-like rhythm (respiratory frequency, fR) during the metabolic acidosis challenge per 5-min window under etonogestrel (red circles, n=18), fluoxetine (orange circles, n=11), and etonogestrel/fluoxetine (purple circles, n=29) exposure. Under etonogestrel/fluoxetine, preparations displayed a powerful potentiation of the respiratory response to metabolic acidosis compared to etonogestrel or fluoxetine alone. *Indicates a significant difference in fR relative to pre-metabolic acidosis values (**p < 0.01, ***p < 0.001, ****p < 0.0001; one-way analysis of variance or Friedman test followed by Benjamini, Krieger and Yekutieli’s multiple comparison test). ^§^Indicates a significant difference between etonogestrel/fluoxetine preparations and etonogestrel or fluoxetine preparations alone (^§§^ p < 0.05; ^§§^p < 0.01 two-way analysis of variance test followed by Benjamini, Krieger and Yekutieli’s multiple comparison test). ∫C4: integrated activity of C4 ventral nerve root; C4: electrical activity of the C4 ventral nerve root.

Preparations from OF1 mice displayed a respiratory-like rhythm in normal-pH of 9.1 ± 0.2 cycles.min^-1^ (n=137) and the classical respiratory response to metabolic acidosis with a respiratory-like rhythm of 14.6 ± 1.0 cycles.min^-1^ (n=14, +40%, p<0.001) as shown in literature (28, 42). As already described in medullary-spinal cord preparations from rodents free from CCHS, etonogestrel (5.10^-2^ µM) increased the respiratory-like rhythm in normal-pH (+11%; n=18, p<0.05), but did not modify the increase in fR induced by metabolic acidosis (+27% vs +40% without the progestin, p=0.11, **Figures 1**, **6B, E, H**) (14, 15, 43).

We first determined the concentration of fluoxetine to be applied as that which does not cause depression of fR. As it has been shown that fluoxetine decreases fR at high concentrations and has no effect at low concentrations in rodent newborns (28, 30, 31), we tested different concentrations: 6.25; 12.5; 25; 50 and 100 µM. At higher concentrations, fluoxetine led to a drop in fR, which in some cases, led to a rhythm arrest (**Figure 6A**). At 25 and 6.25 µM fluoxetine had no significant influence on fR (**Figure 6A**). At 12.5 µM, fluoxetine led to a significant increase in fR (**Figure 6A**). We therefore selected 12.5 µM for rest of the experiments. Then, we observed that the increase in fR induced by metabolic acidosis remained unchanged under fluoxetine (+21% vs +40% without drug, p=0.07, **Figures 1**, **6C, F, H**).

We then co-applied etonogestrel with fluoxetine (**Figure 1**). In normal-pH, fluoxetine did not change the etonogestrel-induced increase in fR (+22% vs 11%, p=0.094), and etonogestrel/fluoxetine led to a potentiation of the respiratory response to metabolic acidosis (+61% vs +40% without drug, p<0.005, **Figures 6D, G, H**).

#### Increasing the 5-HT signaling in Phox2b mutant mice did not have a positive effect

**Figure 7:**
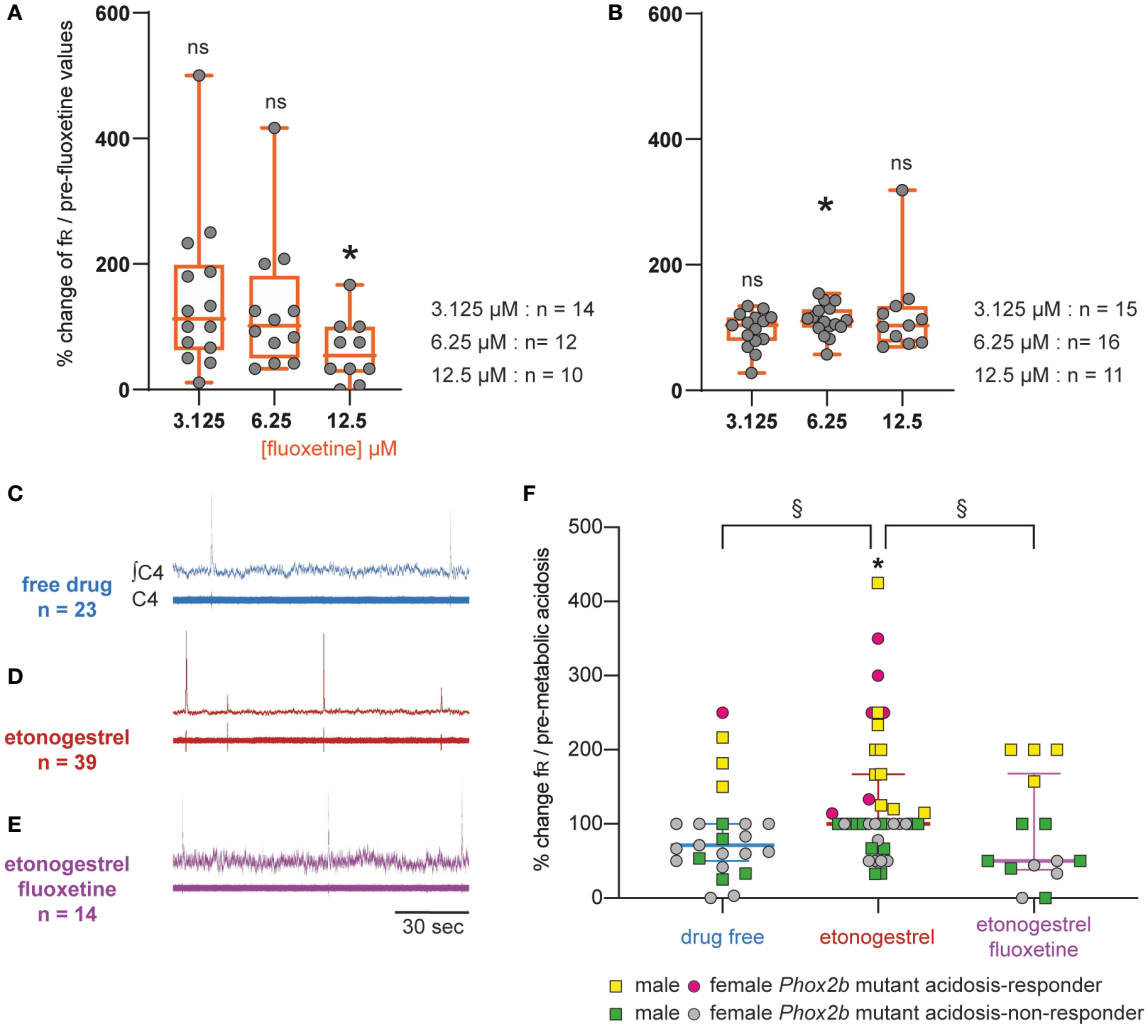
Effect of an increase in 5-HT signaling by fluoxetine in *Phox2b* mutant mice. Scatter plots with a superimposed box and whisker plot (median [Q1; Q3]) showing percentage of change of fR under fluoxetine in *Phox2b* mutants **(A)** and wildtype littermates **(B)**. In *Phox2b* mutants, 3.125 (n=14), 6.25 (n=12) and 12.5 µM (n=10) of fluoxetine. In wildtype littermates, 3.125 (n=15), 6.25 (n=16) and 12.5 µM (n=15) of fluoxetine. * p < 0.05 indicates a significant change in fR relative to pre-fluoxetine values (paired t-test or Wilcoxon matched pairs signed rank test depending on the normality of data distribution). **(C–E)**, Individual phrenic activity traces (fourth cervical ventral nerve root, C4) and integrated C4 activity from medullary-spinal cord from *Phox2b* mutant mice in metabolic acidosis under drug-free (**C**, n=23), etonogestrel (**D**, n=39) and etonogestrel/fluoxetine (**E**, n=14) exposure. **(F)**, Scatter plot with surperimposed median [Q1; Q3] showing the respiratory-like rhythm (respiratory frequency, fR) during the last five minutes of metabolic acidosis in percentage of pre-metabolic acidosis. In the presence of fluoxetine, the restoration of the respiratory response to metabolic acidosis induced by etonogestrel in *Phox2b* mutant mice was abolished. *Indicates a significant change in fR relative to pre-metabolic acidosis values (*p < 0.05; paired t-test). ^§^Indicates a significant difference between the 3 conditions (^§^p < 0.05^;^ Kruskal-Wallis test followed by Benjamini, Krieger and Yekutieli’s multiple comparison test). ∫C4, integrated activity of C4 ventral nerve root; C4, electrical activity of the C4 ventral nerve root. Yellow squares and pink circles represent respectively male and female *Phox2b* mutant mice acidosis-responders (+10% above pre-metabolic values) and green squares and grey circles represent respectively male and female *Phox2b* mutant mice acidosis-non-responders under, drug-free, etonogestrel and etonogestrel/fluoxetine, respectively.

Considering the data obtained in OF1, we applied fluoxetine at a predefined concentration of 12.5 µM in *Phox2b* mutants and wildtype littermates: it caused a significant decrease in fR in *Phox2b* mutants (0.5 ± 0.1 cycles.min^-1^ vs 1.0 ± 0.2 cycles.min^-1^, -37.7%, p=0.02; **Figure 7A**), an effect observed in OF1 when fluoxetine was applied at higher concentrations, and had no significant effect in wildtype littermates (12.8 ± 2.4 cycles.min^-1^ vs 10.8 ± 1.7 cycles.min^-1^, p=0.25; **Figure 7B**). We decreased the concentration to avoid its depressive effect on the respiratory-like rhythm. At 6.25 and 3.125 µM of fluoxetine, we did not observe a depressive effect in *Phox2b* mutant mice (0.8 ± 0.1 and 0.9 ± 0.2 cycles.min^-1^, in 3.125 and 6.25 µM of fluoxetine respectively *vs* 0.8 ± 0.1 and 0.9 ± 0.2 cycles.min^-1^ without fluoxetine; **Figure 7A**) and their wildtype littermates (13.7 ± 2.2 and 10.6 ± 1.4 cycles.min^-1^, in 3.125 and 6.25 µM of fluoxetine respectively *vs* 13.9 ± 2.5 and 9.4 ± 1.0 cycles.min^-1^ without fluoxetine; **Figure 7B**). We decided to apply fluoxetine at 3.125 µM.

Under normal-pH, etonogestrel/fluoxetine led to the loss of the effect of etonogestrel on fR in wildtype littermates (14.0 ± 2.8 vs 13.7 ± 2.2 cycles.min^-1^ free of drugs, p=0.43) and had no effect in *Phox2b* mutants (0.7 ± 0.1 vs 0.8 ± 0.1 cycles.min^-1^, p=0.42). Under metabolic acidosis, opposite to what we expected, the co-application of drugs did not result in potentiation of the respiratory response to metabolic acidosis in wildtype littermates (+45% *vs* +28%, p=0.46) and the restoration of this response observed under etonogestrel alone in *Phox2b* mutants was not present (**Figure 7C, D, E**).

### Characterization of 5-HT metabolic pathways in *Phox2b* mutants, wildtype littermates and OF1 mice

**Figure 8:**
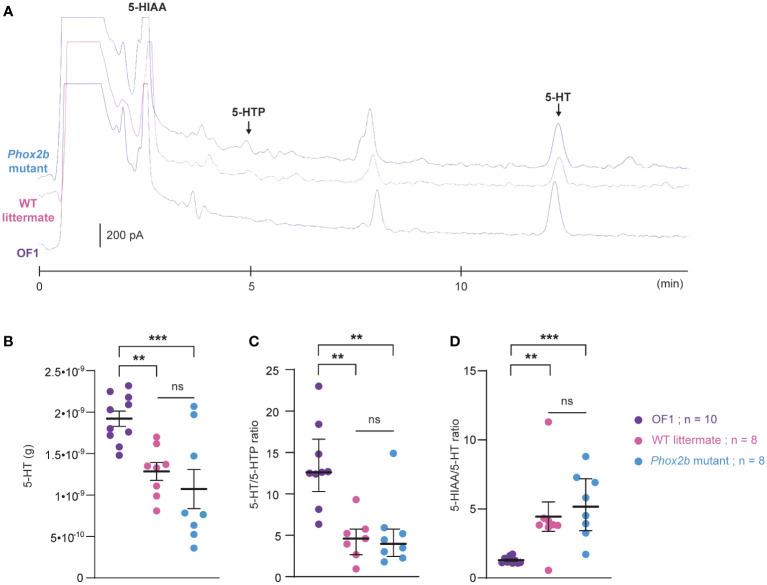
Ultra-high-performance liquid chromatography analysis of 5-HT contents and its related compounds, 5-HTP and 5-HIAA, in the medulla oblongata of *Phox2b* mutants, wildtype littermates and OF1 mice. **(A)**, Example of a chromatogram of medulla oblongata obtained from *Phox2b* mutants (blue; n=8), wildtype littermates (pink; n=8) and OF1 mice (purple; n=10) with indicated peaks of serotonin (5-HT), its precursor 5-hydroxytryptophan (5-HTP) and its metabolite 5-hydroxyindole acetic acid (5-HIAA). **(B)**, Scatter plots showing 5-HT quantity (g of 5-HT per medulla) with superimposed mean ± standard error of the mean. **(C, D)**, Scatter plots showing, respectively, 5-HT/5-HTP and 5-HIAA/5-HT ratio with median [Q1; Q3] superimposed in OF1 (purple filled circles), wildtype littermates (pink filled circles) and *Phox2b* mutants (blue filled circles). * Indicates a significant difference between OF1, *Phox2b* mutants and wildtype littermates (**p < 0.01 and ***p < 0.001; one-way analysis of variance or Kruskal-Wallis test followed by Benjamini, Krieger and Yekutieli’s multiple comparison test) ns, non significant.

We compared the 5-HT metabolic pathways of the medulla oblongata between OF1, *Phox2b* mutants and wildtype littermates (**Figure 8A**) to investigate whether differences could contribute to the observed discrepancy in the respiratory effects of etonogestrel. While medullary 5-HT quantity was lower in *Phox2b* mutants and wildtype littermates than in OF1 (**Figure 8B**), quantities of 5-HTP (5-HT precursor) and 5-HIAA (5-HT degradation product) were higher in wildtype littermates than in OF1, and were similar between *Phox2b* mutants and OF1 mice. The 5-HT/5-HTP ratio was lower in *Phox2b* mutants and wildtype littermates than in OF1 (**Figure 8C**), indicating a weak 5-HT synthesis in *Phox2b* mutants and wildtype littermates. The ratio 5-HIAA/5-HT was higher in *Phox2b* mutants and wildtype littermates than in OF1 (**Figure 8D**), indicating a higher 5-HT turn-over.

## Discussion

Here, in a preclinical model of CCHS, we showed that etonogestrel induces a restoration of the CO_2_/H^+^ chemosensitivity. *Ex vivo* data obtained from medullary-spinal cord preparations suggest that the etonogestrel-induced effects depends on the functional status of medullary serotoninergic systems but not on residual PHOX2B cells in RTN, where structural alterations are considered to be the main cause of central hypoventilation attributed to reduction or loss of CO_2_/H^+^ sensitivity in CCHS patients (5, 6).

### Etonogestrel-induced restoration of CO_2_/H^+^ chemosensitivity in *Phox2b* mutant mice

The present study is the first to report a restoration of CO_2_/H^+^ chemosensitivity in a preclinical model of CCHS and this at various stages of development, confirming previous clinical observations with desogestrel (10). Putting these observations into perspective with the small amount of data available in the literature suggests that etonogestrel either has a specific action compared to progesterone or other progestins, or has enhanced activity compared to progesterone or medroxyprogesterone. As noted the ventilatory response to CO2 of CCHS patients is not increased during pregnancy (12) and medroxyprogesterone, a pregnane progestin, does not improve ventilation in CCHS patients (44).

We observed marked heterogeneity in the response of *Phox2b* mutants to metabolic acidosis, leading to separate the *ex vivo* preparations in acidosis-responders and acidosis-non-responders. Few *Phox2b* mutants were categorized as acidosis-responders in the absence of progestin, consistent with the notion that the absence or dysfunction of PHOX2B RTN cells abolishes or nearly abolishes CO_2_/H^+^ chemosensitivity at this stage of development (5–7, 23). Yet, the very existence of acidosis-responder preparations suggests the possible persistence of H^+^ chemosensitive mechanisms despite *Phox2b* mutations in the RTN. Such a phenomenon could contribute to the residual chemosensitivity described in some CCHS patients (11). Etonogestrel could thus activate or over-activate these residual mechanisms, which would be in line with our observations of both a consistent restoration of the response to metabolic acidosis across all preparations and of an increase in the proportion of acidosis-responders. Whatever the mechanisms involved (etonogestrel-related activation or over-activation of residual RTN sensitivity, or activation of pathways that are silent without progestin), our data show that the *ex vivo* ventilatory-like effects of etonogestrel are not systematically observed, in a manner similar to clinical effects in CCHS patients (10, 13). This suggests that the effects of etonogestrel depend on cell or molecular targets whose functional status is variable among individuals.

At a later stage of development in a whole animal, the stimulatory effect of etonogestrel on CO2/H+ chemosensitivity is also present as indicated by data obtained in the adult mice indicate. This suggests the restoration of chemosensitivity that we observed on ex vivo preparations that do not contain the full capacity of the mechanisms involved in the ventilatory adjustments to CO2/H+ such as inputs arising from peripheral chemoreceptors or supramedullary areas, is robust. Otherwise, on in vivo etonogestrel-treated Phox2b mutant adult mice we observed a stimulating effect of the progestin on the VT during hypercapnia not present in solvent-treated Phox2b mutant mice. This suggests either that the phenomenon observed on ex vivo preparations has an increased potency due to later stage of development and/or the presence of peripheral chemoreceptors and supramedullary structures, or that under these more integrated conditions etonogestrel is likely to activate or over-activate targets other than those present on the ex vivo preparations that we used. Additional experiments are necessary to investigate this question but it is already possible to mention a possible contribution of diencephalic orexinergic systems which, as we have previously shown, can be involved in the restoration of chemosensitivity induced by etonogestrel (15, 43, 45).

### 5-HT neurons, but not PHOX2B residual RTN cells, as a neural basis for the recovery of the CO_2_/H^+^ chemosensitivity in *Phox2b* mutants

Under etonogestrel, the greater number of ROb 5-HT neurons expressing *c-Fos* in acidosis-responder *Phox2b* mutants compared to acidosis-non-responders led us to suppose that 5-HT neurons play a key role. This agrees with previous observations in CCHS-free rodents (14, 45). Many *ex vivo* and *in vivo* studies support the hypothesis that 5-HT neurons within the medullary raphe act as CO_2_/H^+^ chemoreceptors and contribute to a ventilatory response appropriate to maintain homeostasis (37, 38, 46–49). In particular, the activity of ROb 5-HT neurons that heavily innervate respiratory-related structures is increased under hypercapnia, increasing breathing frequency, and potentiating the CO_2_/H^+^ respiratory chemoreflex (46, 49, 50). We therefore believe that, in response to etonogestrel, ROb 5-HT neurons contribute to the restoration of an acidosis response in *Phox2b* mutants through a release of 5-HT within respiratory structures. In this hypothesis, these ROb 5-HT neurons would be more sensitive to an etonogestrel effect in the acidosis-responder individuals. The action of etonogestrel on ROb 5-HT neurons could proceed from their depolarization independently of metabolic acidosis, a depolarization which could then facilitate their stimulation during the acid challenge. This possible stimulating effect of etonogestrel in normopH conditions on 5-HT ROb neurons is in agreement with some of our previous work on WT rodents (14). Alternatively, etonogestrel could directly increase the sensitivity of 5-HT ROb neurons to acidosis. In either case, the molecular target of etonogestrel remains to be identified. Furthermore, it is interesting to recall that 5-HT neurons have been shown to have minimal chemosensitivity at birth (51). It is therefore possible that at later stages of development, such as the adult stage where we found that progestin induced a restoration of the response to hypercapnia in the *Phox2b* mutant to a level comparable to WT littermates, the 5-HT-dependent effect of etonogestrel is more potent. Further experiments are needed to validate this possibility.

Another possible explanation for the restoration of the acidosis-induced increase in respiratory drive could be the activation of RTN residual PHOX2B cells. However, acidosis-responder and acidosis-non-responder preparations did not differ regarding *c-Fos* expression in RTN PHOX2B cells. This result diverges from previous data suggesting that etonogestrel can counteract the loss of function and the toxic effects due to the mutation by modulating the expression of *PHOX2B* and its target genes (16, 17). However, our observation is in agreement with a recent work suggesting that etonogestrel does not affect the neuronal activity through gene transcription within the RTN *i.e.* etonogestrel does not affect the expression of *PHOX2B* or *Gpr4* and *Task2*, the two key pH sensor of PHOX2B RTN cell (52). A possible explanation for the discrepancy between the study conducted on neuroblastoma cells on the one hand and our work and the recent study by Cardani and colleagues on the other hand is that neuroblastoma cell lines express nuclear progesterone receptors, which has not been demonstrated in RTN PHOX2B cells even though hypercapnia-induced RTN *c-Fos* expression is higher in females than males (53). It is important to note that a possible artifact leading to the lack of difference in *c-Fos* expression in PHOX2B cells of the RTN could be related to our experimental conditions *i.e.* only 30 minutes of metabolic acidosis and a temperature of 27°C. However, this hypothesis does not seem very likely since previous work carried out under similar experimental conditions have revealed changes in the expression of *c-Fos* in different respiratory structures of the brainstem including the RTN (14, 15, 21, 35, 54).

### Importance of 5-HT metabolic pathways in the potentiation of the respiratory response to metabolic acidosis induced by co-application of fluoxetine and etonogestrel

An interaction between etonogestrel and 5-HT systems is supported by the fact that potentiation of the response to metabolic acidosis under etonogestrel was only observed, in OF1 mice, when 5-HT systems were boosted by fluoxetine. It can therefore be assumed that the effects of etonogestrel involves excitation of 5-HT neurons leading to 5-HT release, in turn responsible for the enhancement of the respiratory drive. In OF1 mice whose RTN PHOX2B neurons are functional, this hypothesis implies that the etonogestrel-induced 5-HT release would not modulate the respiratory response to metabolic acidosis, which is consistent with published data at this stage of development (38, 55). In contrast, etonogestrel combined with a fluoxetine-induced excess of 5-HT in the synaptic cleft would lead to a reinforcement of the respiratory response to metabolic acidosis, which suggests the need for a large quantity of 5-HT to be released for the respiratory effects of etonogestrel to become visible. In this case, it could be assumed that 5-HT was released in large quantities in *Phox2b* mutant mice to exert a stimulating effect. This would allow for the compensation of the reduced number of RTN PHOX2B cells and/or the mutation-related dysfunction of residual RTN PHOX2B cells. Our ultra-high-performance liquid chromatography data, showing lower 5-HT levels in *Phox2b* mutants than in OF1 individuals, make a larger release of 5-HT in *Phox2b* mutants unlikely. It is possible, as already reported in another context (56), that *Phox2b* mutants have a supersensitivity to 5-HT due to an increased number/functionality of 5-HT receptors. This would allow the etonogestrel-induced 5-HT release to exert a greater stimulating effect on the respiratory network, hence an enhanced fR in metabolic acidosis. It should be noted that since the low quantity of 5-HT was also found in wildtype littermates, the difference between *Phox2b* mutants and the classically used wildtype Swiss OF1 strain did not depend on the mutation but on the strain, as already described (57).

The high potency of 5-HT systems in *Phox2b* mutants, irrespective of its origin, may explain the apparently paradoxical detrimental effect of the co-application of fluoxetine with etonogestrel. Indeed, 5-HT can exert a depressant effect on the respiratory drive at high concentrations (28, 30, 31). Under etonogestrel/fluoxetine co-application, the quantity of 5-HT within the respiratory network could be too high, leading to a depressant effect that cancelled the stimulating effect of etonogestrel. This highlights the importance of the functional status of 5-HT systems for an etonogestrel-induced respiratory effect to occur in a CCHS context. This could explain why some CCHS patients are sensitive to etonogestrel regarding the ventilatory response to CO_2_ while others are not (10, 13). This hypothesis is all the more worth testing given that the known polymorphisms related with serotonergic neurotransmission (58) have been involved in the clinical expression of diseases (59), and the reactions to certain treatments (60). Under this hypothesis, co-administration of etonogestrel with fluoxetine or another selective serotonin reuptake inhibitor could be considered if etonogestrel alone does not produce ventilatory effects, and if 5-HT systems can be characterized as less potent in etonogestrel-insensitive patients compared to etonogestrel-sensitive ones.

To conclude, regardless of the precise molecular pathways involved, collective data point to the potential for targeting 5-HT signaling as a therapeutic intervention associated with etonogestrel for improving the respiratory drive of CCHS patients. Of course, it is important to keep in mind that the data implicating 5-HT systems in the respiratory effects of progestin were collected in a preclinical rodent model at a newborn stage, therefore in conditions far from the clinical observations carried out in CCHS patients. However, the fact that etonogestrel is approved for clinical use for contraception and fluoxetine or other selective serotonin reuptake inhibitors are approved as anti-depressive drugs lends support to translational opportunities.

## Data availability statement

The raw data supporting the conclusions of this article will be made available by the authors, without undue reservation.

## Ethics statement

The animal study was reviewed and approved by Charles Darwin Ethics Committee for Animal Experimentation (Ce5/2011/05; APAFIS#14259-2018032518034654v3 and #2210-2015100812195835v2).

## Author contributions

All authors critically reviewed and approved the manuscript and are accountable its accuracy and integrity. Additionally, AC, contributed substantially to acquisition, analysis, interpretation and discussion of all data, construction of figures and drafting the manuscript. LBi, MR contributed substantially to data acquisition and analysis. FJ, contributed substantially to data acquisition and analysis. RC, contributed substantially to data acquisition and analysis. SP, contributed substantially to data acquisition and analysis. NR, participated in data interpretation. ES, participated in data interpretation. BM, participated in data interpretation. CS, participated in data interpretation and discussed data. TS, participated in data interpretation and discussed data. FC, designed the study and contributed substantially to acquisition, analysis, interpretation and discussion of all data, construction of figures and drafting the manuscript. LBo, obtained funding and designed the study, contributed substantially to acquisition, analysis, interpretation and discussion of all data, construction of figures and drafting the manuscript. All authors contributed to the article and approved the submitted version.

## Ackowledgments

We thank Andrew Lane PhD (Lane Medical Writing) for editing the English text.
